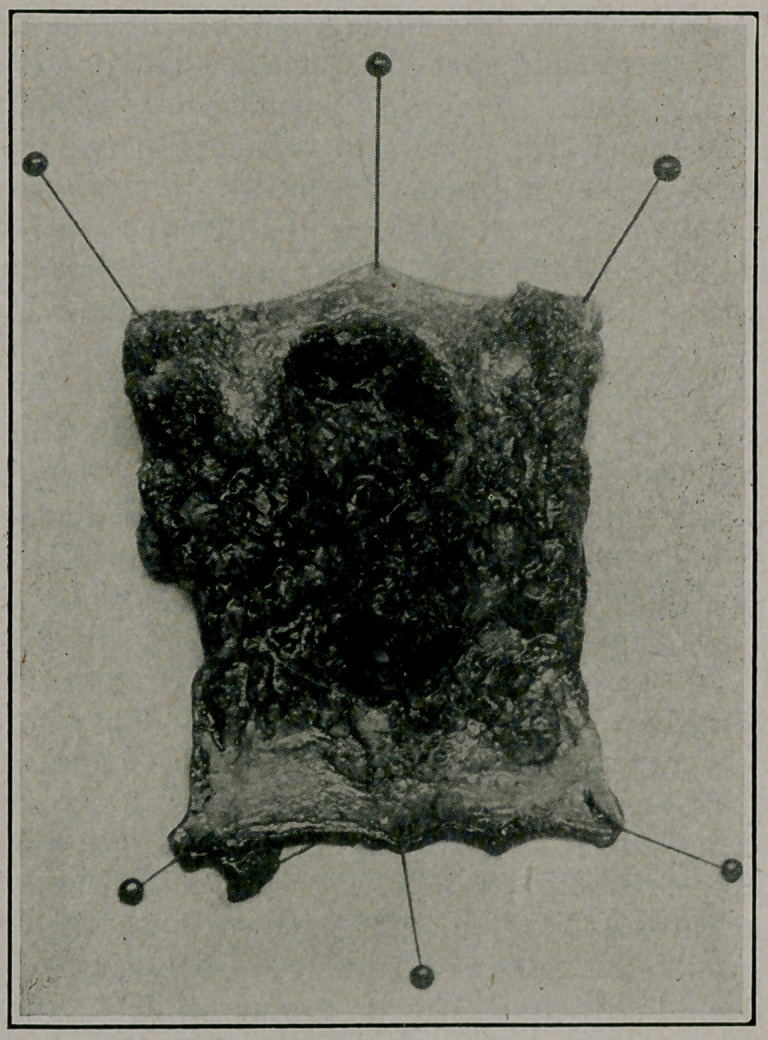# Thoracic Esophagectomy

**Published:** 1913-11

**Authors:** 


Thoracic Esophagectomy. J. Henry Barbat, San Francisco, *Calif. State Jour. of Med.*, June, 1913. (Illustrations reproduced by courtesy of author and editor.) The operation for cancer has been performed about fifty times, with a 100 per cent. mortality. Shock, infection, pneumothorax, interference with the pneumogastric, are the reasons assigned. The pleura and the thoracic organs are less tolerant of handling and of infection than the peritoneum and abdominal organs; moreover, the operation as done at present occupies two or three hours at the least. The author's case survived fifty-two hours, apparently from pneumothorax, a hissing sound being heard with respiration and there being air hunger and cyanosis.

## Figures and Tables

**Figure f1:**
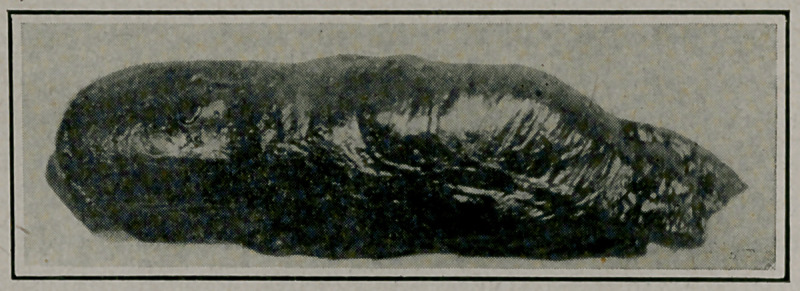


**Figure f2:**